# Salivary Proteome Is Altered in Children With Small Area Thermal Burns

**DOI:** 10.1002/prca.202300107

**Published:** 2025-02-02

**Authors:** Morgan Carlton, Tuo Zang, Tony J. Parker, Chamindie Punyadeera, Joanne Voisey, Leila Cuttle

**Affiliations:** ^1^ School of Biomedical Science Faculty of Health Queensland University of Technology Brisbane Queensland Australia; ^2^ Centre for Children's Burn and Trauma Research Centre for Children's Health Research Queensland University of Technology South Brisbane Queensland Australia; ^3^ Saliva and Liquid Biopsy Translational Research Team Centre for Biomedical Technologies School of Biomedical Sciences Queensland University of Technology Kelvin Grove Queensland Australia; ^4^ The School of Environment and Science Griffith Institute for Drug Discovery (GRIDD) and Menzies Health Institute Queensland (MIHQ) Griffith University Nathan Queensland Australia; ^5^ Centre for Genomics and Personalised Health School of Biomedical Science Faculty of Health Queensland University of Technology South Brisbane Queensland Australia

**Keywords:** biomarkers, burn, paediatric, protein, saliva

## Abstract

Saliva is a child appropriate biofluid, but it has not previously been used to evaluate the systemic response to burn injury in children. The aim of this study was to investigate the salivary proteome of children with small area thermal skin burns relative to different burn characteristics (mechanism, time to re‐epithelialization and risk of emotional distress). SWATH Mass Spectrometry was used to quantify the abundance of 742 proteins in the saliva of children with burns (*n* = 22) and healthy controls (*n* = 37). Eight proteins were differentially abundant in the saliva of children with burns compared to healthy children, and these were associated with immune processes, epidermal cell differentiation and transferrin receptor binding. Eleven proteins were differentially abundant in patients with burns of different mechanisms. Scald burns had an over‐representation of immune/inflammatory response processes, and contact burns had an over‐representation of cornification, intermediate filament assembly and cell death cellular processes. Four proteins were elevated in patients who were at high risk for emotional distress and 15 proteins were correlated with time to wound re‐epithelialization. This pilot study proves that saliva can be used for paediatric biomarker discovery and can be used as a diagnostic and prognostic sample to investigate systemic changes in a paediatric burn cohort.

**Abbreviations**
BCA: bicinchoninic acid
CTSQ: children's trauma screen questionnaire
DDA: data dependent acquisition
FASP: filter aided sample preparation
IAA: iodoacetamide
PEDS‐ES: paediatric emotional distress screener–early screener
PLS‐DA: partial least squares discriminant analysis
SDQ: strengths and difficulties questionnaire
SEIFA: socioeconomic indexes for Australia
STAGE‐tips: stop‐and‐go extraction tips
SWATH: sequential windowed acquisition of all theoretical ion mass
TBSA: total body surface area
TEAB: triethylammonium bicarbonate
VIP: variable importance of projection analysis

## 1 Significance of the Study

This pilot proteomic study demonstrates for the first time that saliva from children with skin burns can be used to identify children at risk of poor outcomes. Children with even small area burns had elevated immune processes and epidermal differentiation proteins associated with burn wound healing detected in their saliva, compared to healthy controls. Saliva proteins were differentially abundant in patients with delayed wound re‐epithelialisation and those at high risk for emotional distress (31% of the cohort studied). After further validation in larger cohorts, these protein biomarkers could be developed into diagnostics to provide early identification of at‐risk patients, changing the clinical trajectory of children with burns to improve patient outcomes.

## 2 Introduction

Burn injury is known to elicit a complex physiological response through the alteration of several biological processes, including the inflammatory response [1, 2, 3], glucose metabolism [4, 5, 6, 7] and other homeostatic processes [8]. Alteration of these responses can lead to an increased risk of developing several diseases, such as cardiovascular disease [9], diabetes [10], and an increased age‐adjusted mortality [11]. In children, burn injuries can also lead to developmental issues, such as stunted growth [12], and bone fragility [13]. Additionally, the trauma associated with burn injury can increase a child's risk of developing psychological disorders in adulthood [14, 15, 16], including post‐traumatic stress disorder [17].

In previous burn research, biological pathways involved in inflammation, wound healing, growth, metabolism and the stress response have been investigated through the targeted analysis of specific biological markers [8]; however, unbiased discovery techniques can provide a better understanding of the biological response to burn injury and help to identify novel monitoring and treatment strategies for patients. In previous paediatric burn research, blood has been the primary sample for biomarker discovery. Blood‐based biomarkers are not ideal when dealing with young children, because blood collection causes unnecessary distress [18], and in outpatient settings, blood is not routinely collected from children. Urine and saliva are underutilized in their prognostic potential for children.

Emerging evidence suggests that the oral micro‐environment reflects systemic changes, and saliva may be a suitable alternative for blood when dealing with a paediatric population [19, 20]. However, the saliva proteome has not previously been investigated in relation to skin burn injury, or specifically for paediatric burn patients. Therefore, the applicability of saliva as a diagnostic and a prognostic medium in this population remains unknown.

We hypothesize that the salivary proteome is different in children with burn versus healthy controls. In this study, semi‐quantitative SWATH Mass Spectrometry proteomic approaches were employed to investigate the salivary proteome of children with burns, compared to a healthy control population. The prognostic potential of saliva for distinguishing between different physical and clinical burn characteristics, including healing outcomes and psychological wellbeing measures, was also examined.

## 3 Materials and Methods

### 3.1 Ethics Approval and Consent to Participate

This research was conducted in accordance with the Declaration of Helsinki. Informed consent was obtained for all participants. Ethics approval to collect data and samples from paediatric burn patients was granted by Children's Health Queensland Hospital and Health Service (#HREC/17/QRCH/270), Queensland Children's Hospital (Site specific approval #SSA/17/QRCH/294) and Queensland University of Technology (QUT) Human Ethics Committees (Administrative approval #1800000026). Ethics to recruit the control cohort was approved by the QUT Human Ethics committee (#1900000038).

### 3.2 Participants

Paediatric patients with a burn injury were recruited from the Pegg Leditschke Children's Burn Centre, Queensland Children's Hospital, Brisbane, Australia between April 2019 and January 2020. Children aged 1–17 years who presented as outpatients were recruited on their second dressing change (6–10 days post‐burn). Prior to administration of any medication, participants provided an unstimulated drool sample [21, 22] and completed the child trauma screening questionnaire if over the age of 8 years. At this time, the parent/caregiver completed a strengths and difficulties questionnaire, and paediatric emotional distress scale early screener regarding the patient (if the child was younger than eight). Families were excluded from participating in the study if (1) the primary caregiver was not present for the wound care procedure, (2) the child or their guardians had difficulty providing informed consent or completing the questionnaires due to insufficient understanding of English or (3) a sufficient drool sample could not be collected.

Healthy children (aged 1–12 years) of a similar demographic profile with no history of burn injury or trauma were recruited from a childcare centre as a control cohort between September 2019 and October 2019. Parents were provided with a list of traumatic events and asked to confirm that their child had not encountered any of these previous traumas. Parents were also asked to complete a demographic questionnaire and strengths and difficulties questionnaire. Following the receipt of written consent, and parent‐completed surveys, a drool sample was collected from each control participant. Participants were excluded from the control cohort if (1) the child had previous trauma, (2) the child had sustained a burn injury in the past, (3) the parent had difficulty providing informed consent or completing the questionnaires due to insufficient understanding of English or (4) a sufficient drool sample could not be collected.

### 3.3 Burn and Participant Characteristics

Demographic information (gender, date of birth, ethnic background, postal area code) for each burn patient was collected verbally or from hospital admission information. For the control participants, parents completed a questionnaire regarding the child's information. Parental socioeconomic status was estimated based on the suburb of the family's primary residence, using the Socio‐Economic Indexes for Areas (SEIFA) Postal Area Index of Relative Socio‐economic Advantage and Disadvantage Tables 2016 [23]. Burn injury data (anatomical location, mechanism, wound depth, percentage of total body surface area burned (%TBSA), time to 95% re‐epithelialization, number of surgeries and first aid treatment) were obtained from medical records. Paediatric emotional distress was assessed in the patient cohort. Parents reported the patient's emotional distress in relation to their burn injury if they were younger than 8 years old, using the Paediatric Emotional Distress Scale—Early Screener (PEDS‐ES) [24]. Children older than 8 years reported their own emotional distress symptoms using the Child Trauma Screening Questionnaire (CTSQ) [15]. A total score ≥8 for the PEDS‐ES [24] and ≥5 for the CTSQ [15] indicated that the child exhibited emotional distress symptoms and was considered at risk for the development of emotional distress symptomology.

### 3.4 Sample Collection

Saliva samples were collected from the burn patient cohort on their second appointment at the Burn Centre, approximately 6–10 days post injury. Saliva samples were collected from the control participants after receiving the completed questionnaires from the parent/guardian. The children were asked to rinse their mouth with water prior to sample collection. Children were asked to drool or spit into a 50 mL conical tube (Thermo Fisher Scientific, MA, USA) by leaning their head forward and collecting the saliva for as long as they were comfortable. If the child was unable to independently provide a sample, a sterile, plastic transfer pipette (Sarstedt, South Australia, Australia) was used to collect the saliva from their mouth and this was dispensed into the conical tube. The passive drool sample was stored on ice or at 4°C until transported to the laboratory for processing, within 6 h of collection [21, 22].

### 3.5 Sample Processing

The passive drool sample was centrifuged at 1500 × *g* for 10 min to remove cell debris, the supernatant transferred into a protein LoBind tube (Eppendorf, Hamburg, Germany), then stored at −80°C until further processing. The salivary protein was precipitated by combining one‐part sample with six‐parts ice‐cold acetone (∼98%; Thermo Fisher Scientific) and incubating at −20°C overnight. The samples were subsequently centrifuged at 10,000 × *g* for 15 min at 4°C, and the protein pellet was resuspended in 50 µL of 0.05 M triethylammonium bicarbonate (TEAB; Sigma‐Aldrich, Missouri, United States). The total protein concentration of each sample was determined using a Pierce BCA protein assay kit (Thermo Fisher Scientific), as per the manufacturer's instructions.

### 3.6 Spectral Library Generation

After all samples were processed, and a small aliquot of each individual sample was put aside for future SWATH analysis, the remainder was used to create pooled samples for the spectral library. A pooled burn saliva sample was created by combining an aliquot of each patient drool sample into a single LoBind tube. This was repeated using the control participant samples to create a pooled control sample. A global pooled sample was created by combining 1.5 mg of both the pooled burn sample and pooled control sample together in a single protein LoBind tube for a total protein amount of 3 mg. This global pooled sample was then fractionated using three different methods: gel electrophoresis, iso electric focusing, and iso‐electric focusing with subsequent gel electrophoresis fractionation (Figure S1).

For fractionation by LDS‐PAGE gel electrophoresis, a 60 µg protein aliquot of the global pooled sample was run on a 4%–12% NuPAGE Bis‐Tris gel (Invitrogen) at 200 V for 25 min. The gel was then fixed, stained for 2 h with Colloidal Coomassie Brilliant Blue Stain (17% ammonium sulphate [Sigma–Aldrich], 3% phosphoric acid, 34% methanol [Sigma–Aldrich] and 0.1% Coomassie Brilliant Blue G‐250 [Bio‐Rad]), and de‐stained overnight in 1% acetic acid (Chem Supply, South Australia, Australia). A sterilized razor blade was used to excise sub‐fractions (*n* = 12) of similar intensity from the global pooled sample lanes using a previous protocol [25]. After each of the 12 sub‐fractions were reduced, alkylated, digested with 10 ng/µL trypsin (Promega, Wisconsin, United States) in 100 mM TEAB, extracted and dried down, they were resuspended in 0.1% formic acid (v/v) for desalting.

For fractionation by iso‐electric focusing, a 750‐µg protein aliquot of the global pooled sample was loaded onto a 3–10 pH linear gradient, 24 cm IPG strip (GE Healthcare, Illinois, United States) and run on an Agilent 3100 OFFGEL fractionator for 64 kV hours, and maintained at 20 µA current until they were removed from the fractionator (Agilent, California, United States). Each iso‐electric focused sub‐fraction (*n* = 24) then underwent filter aided sample preparation (FASP) [26] using a Microcon TM‐30 centrifugal filter device (Merck). After alkylation, digestion with 0.6 µg of trypsin in 0.05 M TEAB, the samples were acidified with formic acid to a final concentration of 0.1% for desalting.

For dual fractionation, the global pooled sample first underwent iso‐electric focusing to generate 24 initial fractions, followed by LDS‐PAGE electrophoresis to further separate these into *n* = 124 total sub‐fractions. Each gel fraction underwent in‐gel digestion as previously described. Desalting for all sub‐fractions was performed using Stop‐and‐go‐extraction tips (STAGE tips) [27] prior to mass spectrometry analysis. The tips were produced in‐house by plugging the end of a 200 µL pipette tip with C18 solid phase extraction membrane. The peptides were dried down and resuspended in 2% ACN in 0.1% FA spiked with index retention time (iRT) peptides (Mimotopes, Victoria, Australia). The global pooled sample fractions were resuspended to a final protein concentration of 0.1, 0.14, and 0.09 mg/mL for the iso‐electric focused fractions, LDS‐PAGE fractions, and the dual fractionated sub‐fractions, respectively. The participant samples were resuspended to a final protein concentration of 0.15 mg/mL.

### 3.7 Mass Spectrometry Analysis

Liquid chromatography tandem mass spectrometry (LC‐MS/MS) was used between January 2021 and July 2021 to perform both data‐dependent acquisition (DDA) and sequential windowed acquisition of all theoretical fragment ion mass (SWATH‐MS) using an Eksigent ekspert 400 nanoLC coupled to a TripleTOF 6600 mass spectrometer (SCIEX, Massachusetts, United States). Samples were randomized and first loaded onto a Trajan Protecol trap column (120 Å, 3 µm, 10 mm × 300 µm) from the autosampler and flushed for 3 min at 10 µL/min (0.1% FA). The flush system was then switched to in‐line, to allow elution of the samples onto the analytical column. The peptides in each sample were separated by an 87‐min gradient method using a C18 nano‐LC resolving column (Eksigent ChromXP C18 3 µm 120 Å (3C18‐CL‐120, 3 µm, 120 Å, 0.3 × 150 mm)) with a flow rate of 5 µL/min across a 68‐min linear gradient of Buffer B (0.1% FA in ACN) from 3% to 25%, followed by a 5‐min linear gradient of 25%–35% mobile phase B. After peptide elution, the column was flushed with 80% mobile phase B for 5 min and re‐equilibrated with 97% Buffer A (0.1% FA) for 8 min before the next injection. For DDA parameters, mass spectrometry survey scans of 0.25‐s in the mass range of 400–1250 m/z were performed, followed by 30 MS/MS scans in the mass range of 100–1500 Da (total cycle time, 1.8 s). SWATH variable window acquisition (SWATH Variable Window Assay Calculator Version 1.1 [SCIEX, March 9, 2020]) with a set of 100 overlapping windows over the range 389.5–1250.5 m/z (1 m/z for the window overlap) was constructed based on precursor m/z frequencies in combined DDA runs. In this method, TOF MS scans were collected over a range of 350–1500 m/z for 0.05 s, then the 100 predefined m/z ranges (SWATH windows) were sequentially subjected to TOF MS/MS scans over the range of 100–1800 m/z and 0.025 s per window resulting in a total duty cycle of 2.6 s.

### 3.8 Library Generation and SWATH Data Extraction

Generation of the protein library and SWATH‐MS data processing was performed using the cloud‐based OneOMICS Suite v3.0 (SCIEX). A peptide identification search was performed for each fractionation method using the built‐in ProteinPilot software (SCIEX) and a Swiss‐Prot generated human protein database (Downloaded from www.uniprot.org/ on September 21, 2020). The database contained only reviewed and non‐redundant proteins. It was concatenated with the common repository of adventitious proteins (cRAP) database (Downloaded from http://www.thegpm.org/crap/ on September 21, 2020) which was used to account for any contaminants common in mass spectrometry work. A ‘.group’ file was generated for each fractionation method (OFFGEL_Lib, LDS‐PAGE_Lib and DualFrac_Lib). In addition, a ‘.group’ file was also created by processing all generated DDA files together (AllFiles_Lib). All four of these library files (.group) were concatenated to create a single large library file for use in subsequent SWATH analysis (Concat_Lib) (Figure S1). Visualization of the SWATH‐MS chromatograms prior to data processing was performed to evaluate the quality of the MS runs. Samples with insufficient profiles that could not be re‐run due to inadequate sample volume, were excluded from analysis (including six patient samples and seven control samples). The false discovery rate (FDR) threshold was set at 10% Global, modified peptides were excluded, the number of peptides per protein was set at 15 and the number of transitions per peptide was set to six [28]. During the extraction of the data, auto‐calibration for RT peptides, a 50‐ppm extracted ion chromatogram extraction width was employed and an 8‐min XIC extraction window was employed. The exported relative protein abundance data were used for the following statistical analyses. The mass spectrometry proteomics data have been deposited to the PRIDE Archive (http://www.ebi.ac.uk/pride/archive/) via the PRIDE partner repository with the data set identifier PXD028078. [29, 30, 31]

### 3.9 Statistical Analysis

To achieve sufficient statistical power for detecting a clinically meaningful difference between groups, a power analysis was conducted based on previous proteomic studies examining proteins in burn blister fluid [32]. With a two‐sided significance level set at 0.05 and a statistical power of 80%, a sample size of *n* = 30 per group was deemed necessary. The raw protein abundance data were normalised using NormalyzerDE online tool (http://quantitativeproteomics.org/normalyzerde) [29, 30, 31] and CycLoess normalization was determined to be the most appropriate method for the data as it provided the lowest pooled coefficient of variation (PCV), pooled median absolute deviation (PMAD) and pooled estimate of variance (PEV) intra‐group variation (Figure S2). The normalized relative protein abundance data were uploaded to MetaboAnalyst 5.0 (https://www.metaboanalyst.ca/) to perform Principal Component Analysis (PCA) and Partial Least Squares Discriminant Analysis (PLS‐DA) to determine the separation between groups. Variable Importance of Projection (VIP) analysis was performed following PLS‐DA to determine the proteins most responsible for the separation between the cohorts. The MetaboAnalyst software will remove proteins with >50% of missing values, and replace the remaining missing data points with a value 1/5 of the minimum positive value for the corresponding variable. In this study, 216 or 0.5% of total values were replaced. No additional filtering or normalization was applied within the MetaboAnalyst software.

Statistical analyses were performed using SPSS v27.0.1.0 (IBM, Sydney, New South Wales, Australia). Pearson Chi‐square tests were used to analyse demographic data between cohorts. General linear regression models were used to determine differences in the mean relative protein abundance between cohorts using the normalized data, and to evaluate differences in protein abundance between subgroups of the patient cohort. Age is known to influence protein levels and was used as a covariate when comparing protein abundance between cohorts. In the patient cohort, both age and time from burn injury to sample collection did not significantly differ between patients with burns from different mechanisms or patients that screened differently for emotional distress and therefore these variables were removed as covariates in the analyses. Pearson correlation analysis was also performed to investigate associations between protein abundance and re‐epithelialization time. Linearity was assessed through manual inspection of the plotted data for all proteins found to be statistically correlated. For all statistical analyses, *p* < 0.05 was considered significant. *p* values were not corrected for multiple testing. Fold change analysis was also performed to identify proteins with ≥2‐fold change, as a biologically meaningful change [33] in protein abundance between cohorts, and for patients with different burn characteristics. Fold change analysis was performed using bootstrapped raw abundance data to allow for the correction of skewness and bias in the distribution of bootstrap estimates and to account for low sample numbers. Bias‐corrected and accelerated (BCa) bootstrapping (*n* = 1000) was applied to the raw protein abundances. Outliers were observed in the data, however, bootstrapping only requires that the sample be representative of the population, therefore these outliers were included in the analysis as large variation in the protein abundance was expected.

Volcano plots were generated to visualize the relationship between proteins found to be statistically different and proteins that differed by ≥2‐fold between groups, using R (https://www.r‐project.org/) and the R package ggplot2. Given the small sample size, *p* values were not corrected for multiple testing as this would increase the likelihood of Type II errors. Gene Ontology (GO) enrichment analysis was performed using Cytoscape v3.8.2 (https://cytoscape.org/) and the BiNGO app (http://apps.cytoscape.org/apps/bingo), for the proteins that were determined to be statistically significant and have ≥ 2‐fold change between cohorts, or sub‐groups of the patient cohort. For these analyses, hypergeometric tests and Benjamini and Hochberg FDR correction were used to determine over‐representation of the GO terms, and the background set was the complete human proteome. The significance threshold was set at 0.05. Only Biological Process GO terms are reported here, as the number of pathways identified for Cellular Component and Molecular Function GO terms were either too large to be meaningful, or there were no significantly enriched GO terms identified from the significant proteins. Simplification of the GO results were performed using the web application REViGO (http://revigo.irb.hr/) to remove redundant GO terms. The simplified results were then visualized using CirGO v2.0 (https://github.com/IrinaVKuznetsova/CirGO).

## 4 Results

### 4.1 Participant Demographics

Thirty‐four paediatric patients with burns and 46 healthy controls were recruited into the study during the limited recruitment timeframe. Some samples had a very low volume (∼1 mL), and once the aliquot had been removed for SWATH analysis, not all samples could be used for the spectral library. The spectral library was created with 28 burn samples (of the 34 recruited, five provided no sample, and one patient withdrew), and 33 control samples (of the 46 recruited, 13 had no volume left). For SWATH analysis, four burn patients had non‐thermal burns (friction of sunburn) and these were removed to try to limit variability, and two samples ran out of volume on MS re‐analysis, leaving 22 burn samples. For the control samples, seven samples did not have enough volume for analysis and two samples ran out of volume on MS re‐analysis, leaving 37 control samples. Participant demographics are reported in Table 1. The burn patient cohort and control cohort had similar gender profiles, however age, ethnicity, and socioeconomic status (as determined by the SEIFA Decile index) significantly differed between the two cohorts (*p* < 0.01). Additionally, both cohorts had similar behavioural attributes, determined by the strengths and difficulties questionnaire. The two cohorts were not pair matched by age, gender, or ethnicity due to the limited timeframe for recruiting control participants, and small numbers of burn patients available for recruitment. Interestingly, the total concentration of protein in the saliva of children in the burn patient group was significantly higher than the control participants (*p* < 0.005). To ensure this did not affect the identification or abundance of protein detected, equal total amounts of protein were loaded for each group in the mass spectrometry analysis. The burn group consisted of patients with small area burns (<3% TBSA), of which 76.2% were superficial partial thickness (SPT), and with equal proportions of scalds (45.5%) and contact (45.5%) burns. Patient burns re‐epithelialized in a median of 9 days, with samples collected between 5‐ and 14‐days post‐burn. Additionally, 31.2% of the patients were characterized as high risk for the development of emotional distress symptoms (Table 1).

**TABLE 1:** Participant demographics legend.

Variable *n* (%)		Burn patient (*n* = 22)	Control (*n* = 37)	Significance (*p* value)
Gender				0.384^c^
	Male	7 (31.8)	16 (43.2)	
	Female	15 (68.2)	21 (56.9)	
Ethnicity				0.002^c^
	Caucasian	13 (59.1)	34 (91.9)	
	Non‐Caucasian	9 (40.9)	3 (8.1)	
SEIFA				<0.001^c^
	Lowest deciles (1–3)	7 (31.8)	24 (64.9)	
	Medium deciles (4–7)	3 (13.6)	11 (29.7)	
	Highest deciles (8–10)	12 (54.6)	2 (5.4)	
Burn depth (*n* = 21^a^)				
	Superficial partial thickness	16 (76.2)	—	
	Deeper than superficial partial thickness	5 (23.8)	—	
Anatomical location				
	Head	2 (9.1)	—	
	Trunk^b^	0 (0.0)	—	
	Groin^b^	0 (0.0)	—	
	Limbs	5 (22.7)	—	
	Hands or feet	10 (45.5)	—	
	Multiple locations	5 (22.7)	—	
Mechanism				
	Flame	2 (9.1)	—	
	Scald	10 (45.5)	—	
	Contact	10 (45.5)	—	
First aid				
	Adequate	19 (86.4)	—	
	Inadequate	3 (13.6)	—	
Risk of emotional distress				
	Low risk	15 (68.2)	—	
	High risk	7 (31.2)	—	
Number of surgeries				
	None	21 (95.5)	—	
	One	1 (4.5)	—	
Median (range)				
Age (years)		6.33 (1.64–13.92)	3.51 (1.65–11.87)	0.008^d^
SDQ emotional problems		1.0 (0.0–9.0)	1.0 (0.0–7.0)	0.202^d^
SDQ conduct problems		1.0 (0.0–6.0)	1.0 (0.0–4.0)	0.941^d^
SDQ hyperactivity		3.5 (0.0–10.0)	3.0 (0.0–10.0)	0.595^d^
SDQ peer problems		0.5 (0.0–4.0)	1.0 (0.0–4.0)	0.280^d^
SDQ prosocial		8.0 (3.0–10.0)	9.0 (5.0–10.0)	0.452^d^
SDQ total difficulties		7.5 (1.0–29.0)	6.5 (0.0–19.0)	0.619^d^
Total protein concentration (µg/mL)		1012.18 (560.71–3607.39)	694.04 (138.39–1478.89)	0.002^d^
TBSA (%)		1 (0.5–3)	—	
Time to re‐epithelialization (days)		9 (6–14)	—	
Time from injury to sample collection (days)		6.4 (5.0–14.0)	—	

Abbreviations: SDQ, strengths and difficulties questionnaire; SEIFA, socio economic indexes for Australia; TBSA, total body surface area.

^a^ Missing data.

^b^ Trunk and Groin burns were included in the study, however, they were accompanied by burns on other anatomical locations and therefore were considered as ‘multiple location’ burns.

^c^ Significance was determined by: Pearson Chi Square test.

^d^ Significance was determined by: Bootstrapped univariate general linear model.

### 4.2 Burn Injury Alters Salivary Protein Abundance

**FIGURE 1:**
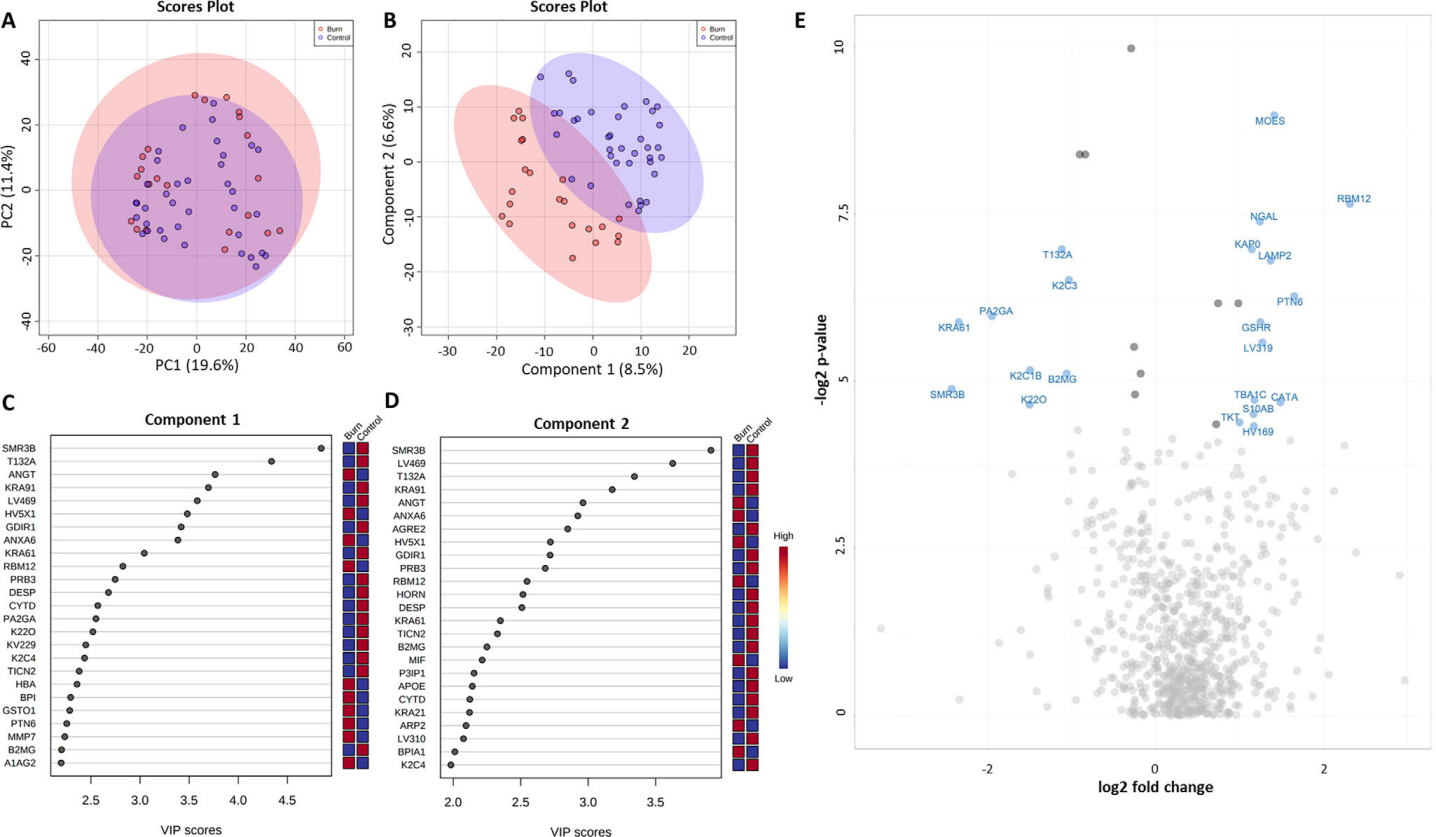
Differential protein analysis of burn and control cohorts. PCA plot did not distinguish between groups (A) whereas PLS‐DA revealed separation of burn patients from healthy controls based on the protein abundance (B); and variable importance in projection (VIP) analysis indicates the 25 proteins most responsible for the separation of cohorts in PLS‐DA analysis in (C) component 1 and (D) component 2. (E) Volcano plot indicating proteins that significantly differed between cohorts and were ≥2‐fold changed in abundance. Labelled proteins significantly differed between the two groups (*p* < 0.05) and were ≥2‐fold higher or lower in burn patients. Light grey dots indicate proteins that were not significantly changed between the two groups, and dark grey dots indicate proteins that were significantly different (*p* < 0.05) but not ≥2‐fold changed between the groups. A1AG2, alpha‐1‐acid glycoprotein 2; AGRE2, adhesion g protein‐coupled receptor E2; ANGT, angiotensinogen; ANXA6, annexin A6; APOE, apolipoprotein E; ARP2, actin‐related protein 2; B2MG, beta‐2‐microglobulin; BPI, bactericidal permeability‐increasing protein; BPIA1, bpi fold‐containing family a member 1; CATA, catalase; CYTD, cystatin‐D; DESP, desmoplakin; GDIR1, rho GDP‐dissociation inhibitor 1; GSHR, glutathione reductase, mitochondrial; GSTO1, glutathione s‐transferase omega‐1; HBA, haemoglobin subunit alpha; HV169, immunoglobulin heavy variable 1–69; HV5×1, immunoglobulin heavy variable 5‐10‐1; HORN, hornerin; K22O keratin, type II cytoskeletal 2 oral; K2C1B keratin, type II cytoskeletal 1B; K2C3 keratin, type II cytoskeletal 3; K2C4 keratin, type II cytoskeletal 4; KAP0, camp‐dependent protein kinase type I‐alpha regulatory subunit; KRA21 keratin, associated protein 2‐1; KRA61 keratin, associated protein 6‐1; KRA91 keratin, associated protein 9‐1; KV229, immunoglobulin kappa variable 2–29; LAMP2, lysosome‐associated membrane glycoprotein 2; LV310, immunoglobulin lambda variable 3–10; LV319, immunoglobulin lambda variable 3–19; LV469, immunoglobulin lambda variable 4–69; MIF, macrophage migration inhibitory factor; MMP7, matrilysin, MOES, moesin; NGAL, neutrophil gelatinase‐associated lipocalin; P3IP1, phosphoinositide‐3‐kinase‐interacting protein 1; PA2GA, phospholipase A2, membrane associated; PRB3, basic salivary proline‐rich protein 3; PTN6, tyrosine‐protein phosphatase non‐receptor type 6; RBM12, RNA‐binding protein 12; S10AB, protein S100‐A11; SMR3B, submaxillary gland androgen‐regulated protein 3B; T132A, transmembrane protein 132A; TBA1C, tubulin alpha‐1C chain; TICN2, testican‐2; TKT, transketolase.

The relative abundances of 742 proteins were quantified in the patient and control saliva samples. Principal component analysis (PCA) failed to distinguish the two cohorts (Figure 1A) however, partial least squares discriminant analysis (PLS‐DA), a supervised cluster analysis, was able to discriminate the two groups with little overlap (Figure 1B). Subsequent variable importance of projection (VIP) analysis of the PLS‐DA data revealed the top 25 proteins responsible for separation of the two groups for component one (Figure 1C) and component two (Figure 1D). *T*‐test comparisons determined that 79 proteins were significantly different between the burn patient and control groups. Following correction for age, only 29 proteins were found to be significantly different between the two cohorts (Table S1). Fold change analysis was performed on bootstrapped (*n* = 1000) mean protein abundance to determine biologically significant changes that were not identified in the pairwise comparisons. As such, 94 proteins were found to be ≥2‐fold increased, and 23 proteins were found to be ≥2‐fold decreased in the burn patient cohort. A volcano plot was created to demonstrate which proteins were significantly different (based on pairwise comparisons) and ≥2‐fold change in the burn group (Figure 1E). In the burn group, 13 proteins were found to be ≥2‐fold increased, while eight proteins were found to be ≥2‐fold decreased in the burn group (Figure 1E).

**FIGURE 2:**
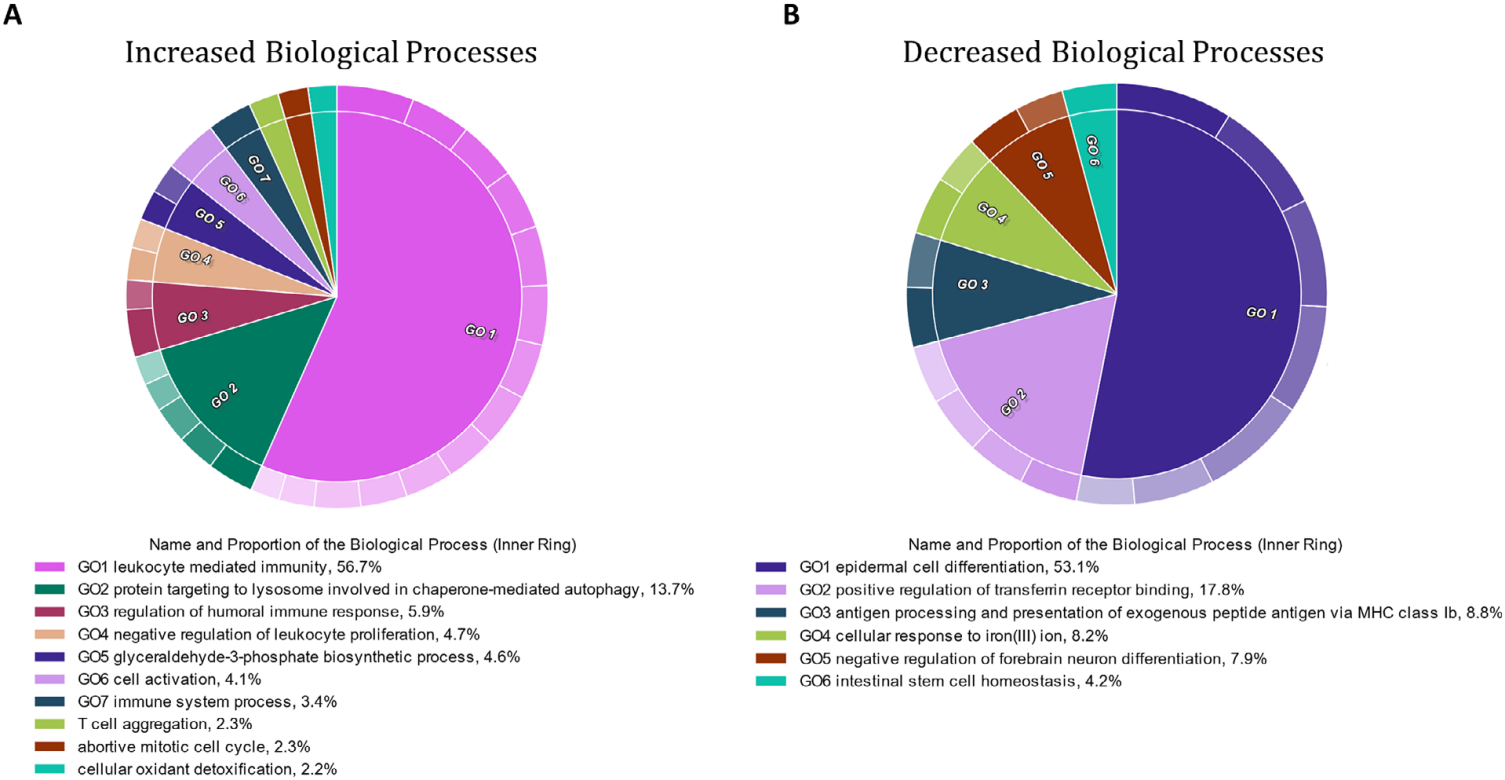
Biological processes relating to inflammation and immune response were increased in the burn cohort. (A) Over‐represented biological process GO terms associated with proteins that had increased abundance in the saliva of paediatric burn patients. (B) Over‐represented biological process GO terms associated with proteins that had decreased abundance in the saliva of paediatric burn patients.

GO analysis was performed to determine the over‐represented biological processes that were associated with the significantly different proteins that had a fold change ≥2 between the two cohorts (Figure 2). The proteins increased in paediatric burn patient saliva returned 39 biological process (BP) GO terms (Figure 2A). Of these, 73% were related to immune processes, including leukocyte mediated immune processes, regulation of humoral immune response and T cell aggregation. Conversely, the proteins found to be decreased in the burn patient saliva returned 19 BP GO terms, which were primarily associated with epidermal cell differentiation (53.1%) and transferrin receptor binding (17.8%) (Figure 2B).

To highlight the proteins that most confidently differed between the two cohorts, the proteins identified in each of the three analysis methods (PLS‐DA VIP, pairwise comparisons and fold change) were compared. Eight proteins were commonly identified in all three analysis methods (Figure S3A). The eight proteins consisted of two that were increased in the saliva of children with burns (tyrosine‐protein phosphatase non‐receptor type 6 and RNA‐binding protein 12), and six that were decreased, including beta‐2‐microglobulin, keratin, type II cytoskeletal 2 oral, keratin‐associated protein 6‐1, submaxillary gland androgen‐regulated protein 3B, phospholipase A2, membrane associated and transmembrane protein 132A (Table 2).

**TABLE 2:** Eight proteins were identified as different between burn patient saliva and healthy saliva using pairwise comparison, fold change analysis and PLS‐DA VIP analysis.

Protein ID	Protein name	Accession #	Abundance in burns
PTN6	Tyrosine‐protein phosphatase non‐receptor type 6	P29350	↑
RBM12	RNA‐binding protein 12	Q9NTZ6	↑
B2MG	Beta‐2‐microglobulin	P61769	↓
K22O	Keratin, type II cytoskeletal 2 oral	Q01546	↓
KRA61	Keratin‐associated protein 6‐1	Q3LI64	↓
PA2GA	Phospholipase A2, membrane associated	P14555	↓
SMR3B	Submaxillary gland androgen‐regulated protein 3B	P02814	↓
T132A	Transmembrane protein 132A	Q24JP5	↓

### 4.3 Scald and Contact Burns Have Different Salivary Protein Abundance Profiles

**FIGURE 3:**
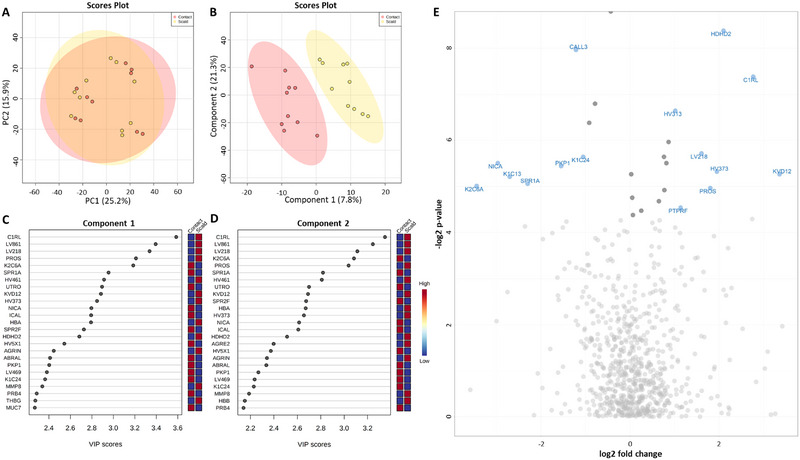
Differential protein analysis of scald and contact burns. PCA plot did not distinguish between groups (A), however, PLS‐DA revealed separation of burn patients based on mechanism (B); and variable importance in projection (VIP) analysis indicates the 25 proteins most responsible for the separation of the two groups in PLS‐DA analysis in (C) component 1 and (D) component 2. (E) Volcano plot indicating proteins that significantly differed between cohorts and were ≥2‐fold changed in abundance. Labelled proteins significantly differed between the two groups (*p* < 0.05) and were ≥2‐fold higher or lower in burn patients. Light grey dots indicate proteins that were not significantly changed between the two groups, and dark grey dots indicate proteins that were significantly different (*p* < 0.05) but not ≥2‐fold changed between the groups. ABRAL, costars family protein ABRACL; AGRE2, adhesion g protein‐coupled receptor E2; AGRIN, agrin; C1RL, complement C1r subcomponent‐like protein; CALL3, calmodulin‐like protein 3; HBA, haemoglobin subunit alpha; HBB, haemoglobin subunit beta; HDHD2, haloacid dehalogenase‐like hydrolase domain‐containing protein 2; HV313, immunoglobulin heavy variable 3–13; HV373, immunoglobulin heavy variable 3–73; HV461, immunoglobulin heavy variable 4–61; HV5×1, immunoglobulin heavy variable 5‐10‐1; ICAL, calpastatin; K1C13 keratin, type I cytoskeletal 13; K1C24 keratin, type I cytoskeletal 24; K2C6A keratin, type II cytoskeletal 6A; KVD12, Immunoglobulin kappa variable 1D‐12; LV218, Immunoglobulin lambda variable 2–18; LV469, immunoglobulin lambda variable 4–69; LV861, immunoglobulin lambda variable 8–61; MMP8, neutrophil collagenase; MUC7, mucin‐7; NICA, nicastrin; PKP1, plakophilin‐1; PRB4, basic salivary proline‐rich protein 4; PROS, vitamin K‐dependent protein S; PTPRF, receptor‐type tyrosine‐protein phosphatase F; SPR1A, cornifin‐A; SPR2F small proline‐rich protein 2F, THBG, thyroxine‐binding globulin; UTRO, utrophin.

Only scald and contact mechanism burns were investigated as there were insufficient numbers of patients with flame burns for analysis. PCA analysis did not discriminate between the groups (Figure 3A) but PLS‐DA analysis revealed a distinct separation between the scald and contact groups (Figure 3B). The top 25 proteins responsible for this separation along each component were determined through VIP analysis (Figure 3C,D) and 27 distinct proteins were responsible, 23 were responsible for separation in both components. Pairwise comparison between the saliva of children with scald burns and contact burns revealed 27 proteins were significantly different between the two groups (Table S2). Of the 27 significantly different proteins, 16 were elevated in scald burns, while 11 were elevated in contact burns. Fold change analysis revealed that 130 proteins had an abundance that differed by ≥2‐fold between the groups. Specifically, 52 proteins were elevated in the group with contact burns and 78 proteins were elevated in scald burns. The generation of a volcano plot was used to identify which protein abundances were statistically different and ≥2‐fold changed between the groups (Figure 3E). As such, it was determined that eight proteins were increased in the saliva of the scald group compared to the contact burn group and seven proteins were increased in the saliva of the group with contact burns, compared to those with scald burns.

**FIGURE 4:**
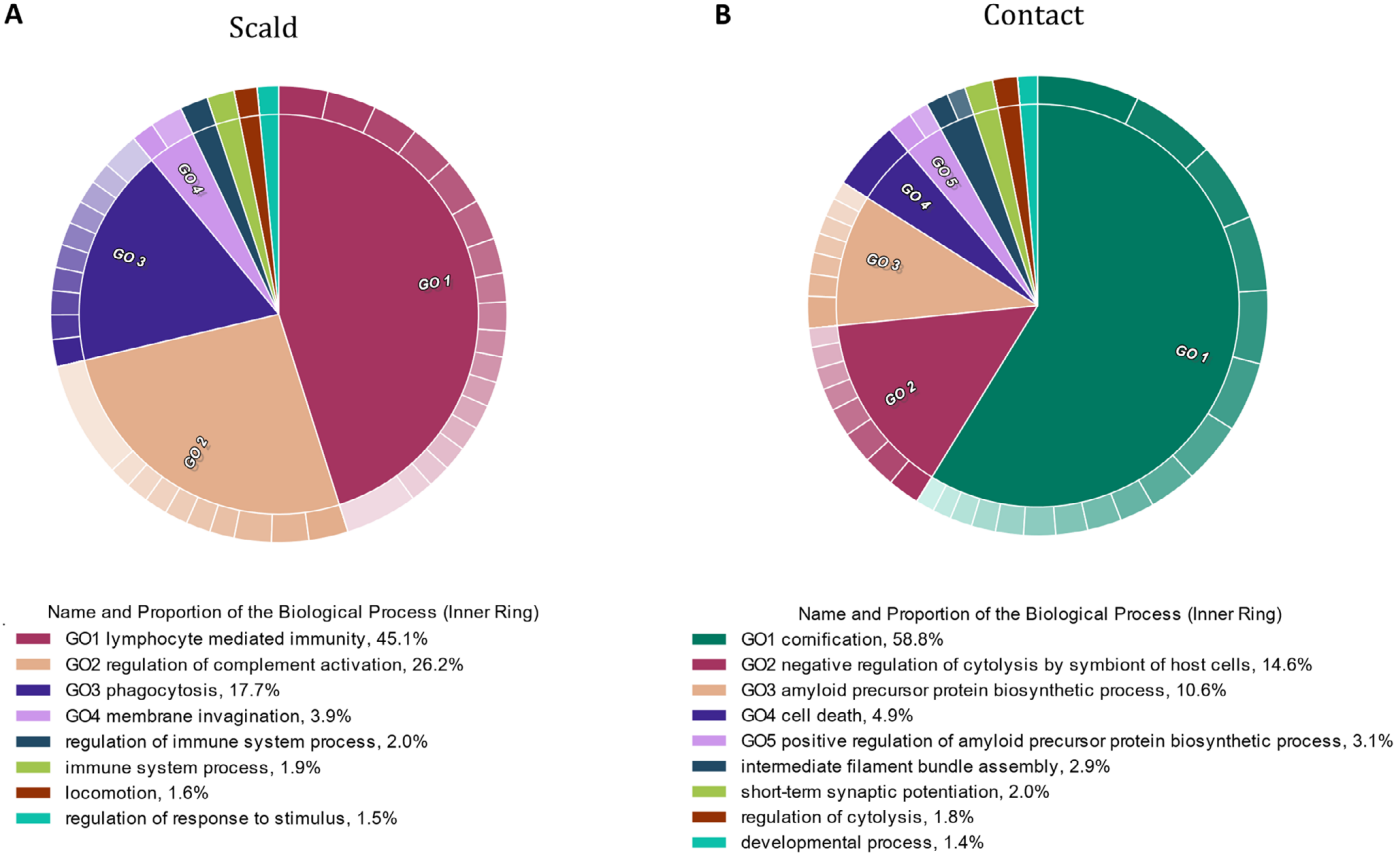
Proteins elevated in patients with scald burns and contact burns were primarily associated with inflammation or immune response and cell death, respectively. (A) Over‐represented biological process GO terms associated with proteins increased in the saliva of patients with scald burns. (B) Over‐represented biological process GO terms associated with proteins with increased abundance in the saliva of patients with contact burns.

GO analysis was performed using these proteins to determine the over‐represented biological processes associated with the proteins (Figure 4). GO analysis of the proteins increased in scald burns (Figure 4A) revealed that 96.8% of returned GO terms were associated with the immune/inflammatory response. More specifically, 45.1% of the GO terms were related to lymphocyte mediated immunity, 26.2% related to the complement cascade, and 21.6% related to phagocytosis and plasma membrane invagination. The other non‐immune related GO terms were associated with locomotion and response to stimuli. Conversely, GO analysis of the proteins increased in patients with contact burns (Figure 4B) revealed that biological processes relating to cornification (58.8%), amyloid precursor protein biosynthetic processes (21.3%) and cell death/cytolysis (13.7%) were primarily over‐represented.

To highlight the proteins that most confidently differed between the two cohorts, the proteins identified in each of the three analysis methods (PLS‐DA VIP, pairwise comparisons and fold change) were compared. In doing so, 11 proteins were identified in all three analysis methods (Figure S3B). These included six proteins that were increased in the saliva of children with scald burns and five that were increased in the saliva of children with contact burns (Table 3).

**TABLE 3:** Eleven proteins were robustly identified to have differential abundance between the saliva of children with scald burns compared to those with contact burns.

Protein ID	Protein name	Accession #	Increased in:
HDHD2	Haloacid dehalogenase‐like hydrolase domain‐containing protein 2	Q9H0R4	Scald
C1RL	Complement C1r subcomponent‐like protein	Q9NZP8	Scald
LV218	Immunoglobulin lambda variable 2–18	A0A075B6J9	Scald
HV373	Immunoglobulin heavy variable 3–73	A0A0B4J1V6	Scald
KVD12	Immunoglobulin kappa variable 1D‐12	P01611	Scald
PROS	Vitamin K‐dependent protein S	P07225	Scald
K1C24	Keratin, type I cytoskeletal 24	Q2M2I5	Contact
NICA	Nicastrin	Q92542	Contact
PKP1	Plakophilin‐1	Q13835	Contact
SPR1A	Cornifin‐A	P35321	Contact
K2C6A	Keratin, type II cytoskeletal 6A	P02538	Contact

### 4.4 Several Proteins Were Significantly Correlated with Time to Re‐Epithelialization

In the burn patient group, the time from injury to 95% re‐epithelialization of the burn wound was a median of 9 days, ranging from 6 to 14 days. Correlation analysis was performed to determine whether proteins were associated with the re‐epithelialization time for the burns. For this analysis, one participant was removed due to requiring a skin grafting operation, which altered the healing trajectory. The analysis revealed that 15 proteins were significantly correlated with the time to re‐epithelialization (Table 4). Specifically, nine proteins were negatively correlated with the time to re‐epithelialization, and indicative of healing, while six were positively correlated, and indicative of delayed healing. Evaluation of the *r* value for each protein revealed that all proteins exhibited a fair correlation (*r* = 0.4–0.6 [34]) with re‐epithelialization time. GO analysis was performed on these proteins; however, no over‐represented biological processes were identified.

**TABLE 4:** Fifteen proteins were correlated with the time it took for patients to reach 95% re‐epithelialization of the burn wound (*n* = 21).

Protein ID	Protein name	Accession #	*R* value^a^	*p* value^b^
*Negatively correlated*				
CPNS1	Calpain small subunit 1	P04632	−0.562	0.008
TBA1C	Tubulin alpha‐1C chain	Q9BQE3	−0.545	0.011
RBM12	RNA‐binding protein 12	Q9NTZ6	−0.523	0.015
AGRG2	Adhesion G‐protein coupled receptor G2	Q8IZP9	−0.487	0.025
LCN15	Lipocalin‐15	Q6UWW0	−0.484	0.026
TPM3	Tropomyosin alpha‐3 chain	P06753	−0.478	0.028
NECT4	Nectin‐4	Q96NY8	−0.466	0.033
SUMF2	Inactive C‐alpha‐formylglycine‐generating enzyme 2	Q8NBJ7	−0.458	0.037
NAGK	N‐acetyl‐D‐glucosamine kinase	Q9UJ70	−0.453	0.039
*Positively Correlated*				
KLK10	Kallikrein‐10	O43240	0.525	0.014
TSN6	Tetraspanin‐6	O43657	0.473	0.030
IGJ	Immunoglobulin J chain	P01591	0.457	0.037
APOD	Apolipoprotein D	P05090	0.446	0.042
KVD12	Immunoglobulin kappa variable 1D‐12	P01611	0.440	0.046
ITIH3	Inter‐alpha‐trypsin inhibitor heavy chain H3	Q06033	0.434	0.049

^a^ *R* value determined using Pearson correlation.

^b^ *p* value determined from Pearson correlation.

### 4.5 Four Proteins Were Elevated in Saliva of Patients at High Risk for Developing Emotional Distress Symptoms

**FIGURE 5:**
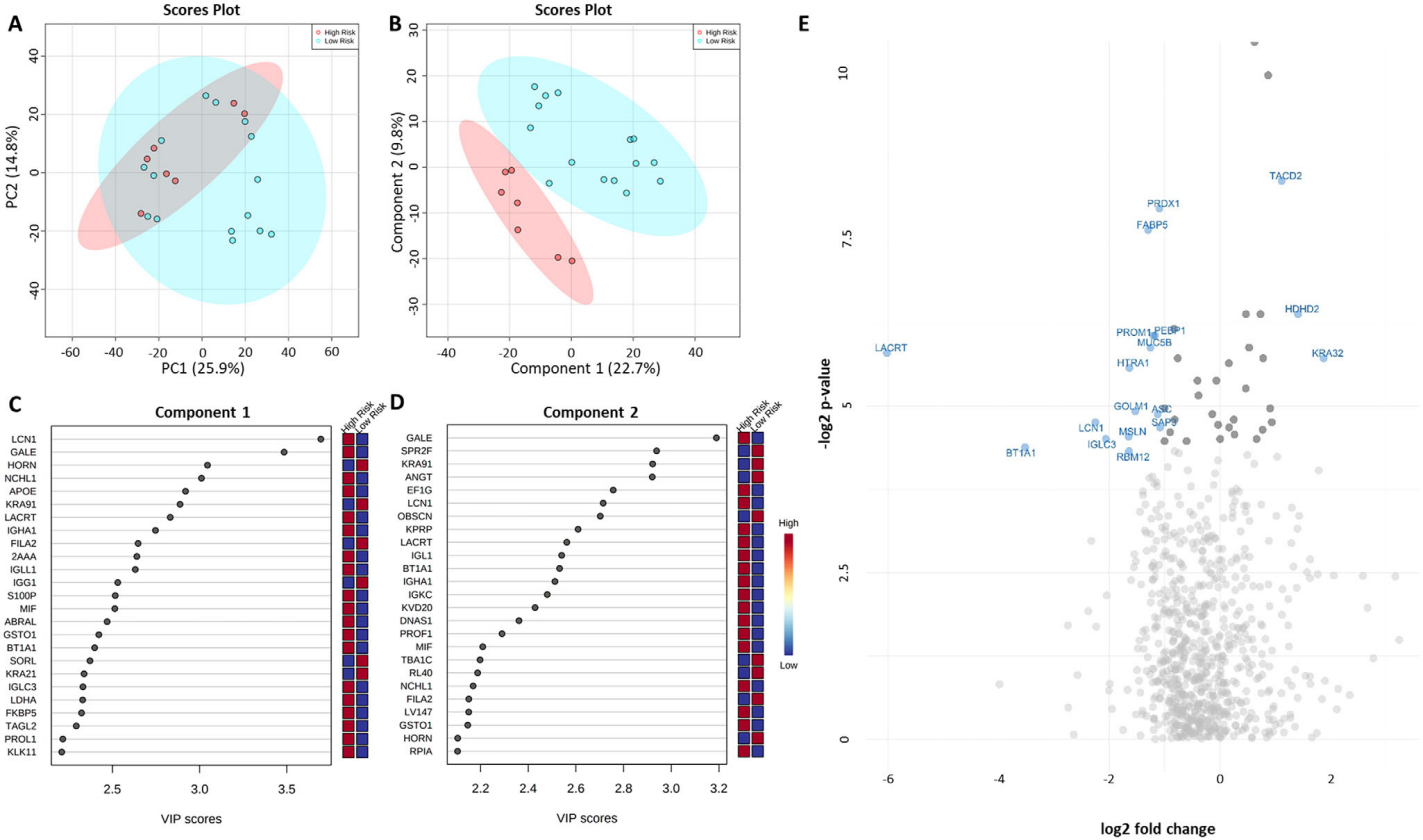
Differential protein analysis of patients who screened low and high risk for emotional distress. PCA plot did not distinguish between groups (A), however, PLS‐DA revealed separation of burn patients based on emotional distress; and variable importance in projection (VIP) analysis indicates the 25 proteins most responsible for the separation of the two groups in PLS‐DA analysis in (C) component 1 and (D) component 2. (E) Volcano plot indicating proteins that significantly differed between high and low emotional distress patients and were ≥2‐fold changed in abundance. Labelled proteins significantly differed between the two groups (*p* < 0.05) and were ≥2‐fold higher or lower in burn patients. Light grey dots indicate proteins that were not significantly changed between the two groups, and dark grey dots indicate proteins that were significantly different (*p* < 0.05) but not ≥2‐fold changed between the groups. 2AAA, serine/threonine‐protein phosphatase 2A 65 kDa regulatory subunit a alpha, isoform; ABRAL, costars family protein ABRACL; ANGT, angiotensinogen; APOE, apolipoprotein E; ASC, apoptosis‐associated speck‐like protein containing a card; BT1A1, butyrophilin subfamily 1 member A1; DNAS1, deoxyribonuclease‐1; EF1G, elongation factor 1‐gamma; FABP5, fatty acid‐binding protein 5; FILA2, filaggrin‐2; FKBP5, peptidyl‐prolyl cis‐trans isomerase FKBP5; GALE, udp‐glucose 4‐epimerase; GOLM1, golgi membrane protein 1; GSTO1, glutathione s‐transferase omega‐1; HDHD2, haloacid dehalogenase‐like hydrolase domain‐containing protein 2; HORN, hornerin; HTRA1, serine protease; IGG1, immunoglobulin gamma‐1 heavy chain; IGHA1, immunoglobulin heavy constant alpha 1; IGKC, immunoglobulin kappa constant; IGL1, immunoglobulin lambda‐1 light chain; IGLC3, immunoglobulin lambda constant 3; IGLL1, immunoglobulin lambda‐like polypeptide 1; KLK11, kallikrein‐11; KPRP, keratinocyte proline‐rich protein; KRA21, keratin‐associated protein 2‐1; KRA32, keratin‐associated protein 3‐2; KRA91, keratin‐associated protein 9‐1; KVD20, immunoglobulin kappa variable 3D‐20; LACRT, extracellular glycoprotein lacritin; LCN1, lipocalin‐1; LDHA, L‐lactate dehydrogenase a chain; LV147, immunoglobulin lambda variable 1–47; MIF, macrophage migration inhibitory factor; MSLN, mesothelin; MUC5B, mucin‐5B; NCHL1, neural cell adhesion molecule l1‐like protein; OBSCN, obscurin; PEBP1, phosphatidylethanolamine‐binding protein 1; PRDX1, peroxiredoxin‐1; PROF1, profilin‐1; PROL1, opiorphin prepropeptide; PROM1, prominin‐1; RL40, ubiquitin‐60s ribosomal protein L40; RPIA, ribose‐5‐phosphate isomerase; S100P, protein S100‐P; SAP3, ganglioside gm2 activator, SORL, sortilin‐related receptor; SPR2F, small proline‐rich protein 2F; TACD2, tumour‐associated calcium signal transducer 2; TAGL2, transgelin‐2; TBA1C, tubulin alpha‐1C chain.

Comparative analyses were performed to identify differences in the abundance of salivary proteins between children with burns that screened high‐risk for the development of emotional distress symptoms and those that screened low risk. Unsupervised PCA analysis showed a complete overlap of both sub‐groups (Figure 5A); however, tight clustering indicated less variation between the samples of the high‐risk group compared to the low‐risk group. PLS‐DA exhibited complete separation of the two groups, with the high‐risk group maintaining its more limited variance (Figure 5B). VIP analysis of the PLS‐DA data indicated the top 25 proteins responsible for the separation of the two groups in both component 1 (Figure 5C) and component 2 (Figure 5D). Further examination of the protein lists revealed that 39 distinct proteins were responsible for the separation (14 unique to component 1 only, 14 unique to component 2 only and 11 in both components). Pairwise comparisons revealed that 45 proteins were statistically different between children with burns that screened high‐risk for the development of emotional distress symptoms and those that screened low risk (Table S3). Specifically, 25 proteins were significantly elevated in the low‐risk group, while 20 were significantly elevated in the high‐risk group. Subsequently, fold change analysis was performed on bootstrapped mean raw abundance data. It was determined that 120 proteins were ≥2‐fold increase in high‐risk group, while 49 proteins ≥2‐fold increase in low‐risk group. A volcano plot was generated to determine which proteins were significantly elevated and 2‐fold different between the two risk groups (Figure 5E). It was determined that three proteins were significantly elevated by more than 2‐fold in the low‐risk group, while 14 proteins were significantly elevated by ≥2‐fold in the high‐risk group.

Comparison of the results from the three analysis methods resulted in the identification of four proteins that were highlighted as proteins that differed between the two groups with most confidence (Figure S3C). All four of the highlighted proteins were found to be increased in the high‐risk group (Table 5).

**TABLE 5:** Four proteins were determined to be elevated in patients that screened as high risk for emotional distress, using three methods of analysis.

Protein ID	Protein name	Accession #	Increased in:
LACRT	Extracellular glycoprotein lacritin	Q9GZZ8	High risk
LCN1	Lipocalin‐1	P31025	High risk
IGLC3	Immunoglobulin lambda constant 3	P0DOY3	High risk
BT1A1	Butyrophilin subfamily 1 member A1	Q13410	High risk

## 5 Discussion

This study is the first to investigate the salivary protein profile of paediatric burn patients and evaluate the efficacy of saliva for detecting changes in biological processes associated with burn injury. Saliva is an ideal biofluid for children as it is both non‐invasive and non‐traumatic to collect [35, 36, 37]. Aside from oral maladies, such as dental caries, changes induced by other medical conditions in the salivary proteome of children have not been previously evaluated.

The comparison of salivary abundance of specific proteins in children with burn injury and healthy control children in this study identified eight proteins that differed between the two cohorts. Interestingly, all eight proteins have not previously been investigated in burn patients before. The two proteins that were found to be increased in the saliva of children with burns were tyrosine‐protein phosphatase non‐receptor type 6 (PTPN6) and RNA‐binding protein 12 (RBM12). PTPN6 is an enzyme and pro‐angiogenic protein that has been reported to restore the proliferative ability of endothelial cells following TIMP‐2 inhibition [38]. It also inhibits pro‐inflammatory cytokines [39] and mediates neutrophil infiltration [40]. The functions of RBM12, the other protein found to be increased in the burn patient saliva, are largely unknown, apart from its role in RNA binding.

The six proteins found to be significantly decreased in the burn patient saliva were beta‐2‐microglobulin (B2M), keratin, type II cytoskeletal 2 oral (K22O), keratin‐associated protein 6‐1 (KRA61), membrane associated phospholipase A2 (PA2GA), submaxillary gland androgen‐regulated protein 3B (SMR3B) and transmembrane protein 132A (T132A). Very little research has investigated the role of T132A, KRA61, K22O, and SMR3B, which makes it difficult to comment on why they are decreased in the saliva of burn patients. B2M is a cell surface protein involved in the presentation of peptide antigens to the immune system [41]. A decrease in the abundance of this protein may indicate a reduction in antigen‐presenting processes in the burn cohort, or that there was increased relative B2M abundance in the control group, who were recruited from a childcare setting, where infection is known to be increased [42, 43]. Unfortunately, it is unknown whether the children of the control group had recently recovered from or were currently experiencing a respiratory or other infection at the time of sample collection, which is a limitation of this study. PA2GA is a pro‐inflammatory membrane protein which has previously been reported to be increased in the plasma of fatally wounded burn patients [44], patients who had shock or multiple organ failure [45] or if their burns were infected [46]. The patients involved in this study had non‐severe burns and therefore, it might be anticipated that this protein would not be elevated in this study.

Burn mechanism is an important consideration for determination of treatment options [47]; however, limited research has been performed to compare the underlying biology of different burn mechanisms. In this study, several proteins were identified to be differentially abundant in saliva of patients with scald and contact burns. The six proteins elevated in the saliva of scald patients included haloacid dehalogenase‐like hydrolase domain‐containing protein 2 (HDHD2), complement C1r subcomponent‐like protein (C1RL), immunoglobulin lambda variable 2–18 (LV218), immunoglobulin heavy variable 3–73 (HV373), immunoglobulin kappa variable 1D‐12 (KVD12) and vitamin‐k dependent protein S (PROS). LV218, HV373 and KVD12, are immune related proteins [48], and C1RL and PROS are involved in inflammation [49, 50]. HDHD2 has a role in glycation repair [51], and glycation within the skin can lead to reduced elasticity, increased scarring and delayed wound closure [52]. Additionally, the GO analysis of the significantly more abundant biological processes associated with scald burns highlighted inflammatory and immune responses were more prevalent.

Conversely, the five proteins elevated in the saliva of children with contact burns were Keratin, type I cytoskeletal 24 (K1C24), Nicastrin (NICA), Plakophilin‐1 (PKP1), Cornifin‐A (SPR1A) and Keratin, type II cytoskeletal 6A (K2C6A). K1C24 and K2C6A are both intermediate filament proteins, that have roles in cell growth, migration and death; NICA is an enzyme that regulates the Pi3K/AKT pathway through the cleavage of notch; PKP1 is a desmosomal protein that is involved in regulating keratinocyte proliferation; and SPR1A is involved in cellular migration during wound healing [53] and the cornification of keratinocytes [54, 55]. These proteins are primarily involved in cellular regulation, potentially indicating that the biology underlying contact burns is directed toward an epithelial remodelling and wound healing response. This was also observed in the GO analysis, where the results for the contact burns revealed over‐representation of cellular processes, specifically those of cornification, intermediate filament assembly and cell death.

Many of the proteins that were negatively correlated with time to re‐epithelialization (improved healing) have roles in stimulating cellular proliferation, migration, or adhesion. Calpain small subunit 1 (also known as calpain 4), was the most significant negatively correlated protein with time to re‐epithelialization and it alters epithelial cell migration through regulating expression of extracellular galectin‐3 [56, 57]. Similarly, tubulin alpha chain 1C (TUBA1C), adhesion G‐protein coupled receptor G2 (ADGRG2), and nectin‐4 have been associated with cell migration and proliferation, albeit in cancer cell types [58, 59, 60, 61]. N‐acetyl‐D‐glucosamine kinase is also known to increase migration through interaction with dynein motor complex [62]. Tropomyosin 3 (TPM3) was also identified to be negatively correlated with re‐epithelialization time and may also impact on healing through regulating the speed and efficiency of epithelial cell migration [63]. Tropomyosin has many different isoforms [64, 65] and the data presented here may suggest that TPM3 isoforms are involved in efficient cell migration. The other three proteins negatively correlated with re‐epithelialization time include RBM12, lipocalin‐15 and inactive C‐alpha‐formylglycine‐generating enzyme 2 (SUMF2), but there is a lack of wound healing research for these proteins so their relationship with re‐epithelialization time is unknown.

Proteins that were found to be positively correlated with re‐epithelialization time (delayed healing) are involved in the immune response, such as immunoglobulin kappa variable 1D‐12 and immunoglobulin J chain, or impede cell migration or proliferation. Kallikrein‐10 is a tumour suppressor that decreases cellular proliferation and induces apoptosis [66, 67]. Increased levels of Tetraspanin‐6 (TSPAN6) directs syntenin and syndecan‐4 proteins toward lysosomal degradation [68], resulting in decreased binding of to the ECM, where they are involved in cell adhesion and migration [69]. Apolipoprotein D (APOD) has a primary role in lipid transport but has been found to be involved in stress‐induced inflammation [70], and may be an inhibitor of cell growth [70], as APOD is inhibited by estrogens, which stimulate cell growth, and induced by androgens, which inhibit cell growth. Inter‐alpha‐trypsin inhibitor heavy chain H3 is a peptide that is involved in the stabilization of the ECM, through binding with two other peptides. It is also involved in binding hyaluronic acid during inflammation [71].

Many of the proteins previously described as relating to wound healing processes have been reported in cells directly within the wound, and therefore it is interesting that in this study they have also been detected in saliva. It is unknown whether the proteins found to be associated with healing response in the saliva are present as a side effect of systemic healing processes that are initiated in response to a wound and would also be present in the blood. Another alternative is that these proteins are stimulated in saliva so that topical application to the wound can mediate wound healing. Some studies suggest that the proteins and growth factors within saliva may have a beneficial effect on epithelial wound healing [72, 73] and this may be the reason why animals instinctively lick their wounds [74, 75]. However, more research is still required to fully understand the relationship between proteins in the blood and saliva, and the effect of saliva on wound healing as a topical therapeutic agent.

Four salivary proteins were identified to be different between paediatric burn patients with low risk of developing emotional distress symptomology, and patients at high risk. The four identified proteins included extracellular glycoprotein lacritin (LACRT), lipocalin‐1 (LCN‐1), immunoglobulin lambda constant 3 (IGLC3) and butyrophilin subfamily 1 member A1 (BT1A1), all of which were found to be increased in the high‐risk group. Interestingly, LACRT and LCN‐1 are both proteins found primarily in tears, but have been identified in other biological fluids, including saliva [76, 77]. Specifically, LACRT is secreted from the submandibular salivary gland [78] and has been associated with inflammatory disorders such as dry eyes [79], while LCN‐1 is secreted from the lingual von Ebner's glands [76]. Salivary LCN‐1 was previously reported to increase in pigs placed under stressful conditions (i.e., snaring restraint) [80]. LCN‐1 is co‐localized with adrenocorticotropic hormone in the pituitary gland [81], which may further suggest that it has a role in the stress response. IGLC3 is an immune protein, and BT1A1 is a membrane protein primarily found in milk fat globules [82] that has also been found to regulate the immune response in vivo [83]. The relationship of the other three proteins with emotional distress has not been elucidated.

An interesting observation in this study was that the total concentration of protein in the saliva of children in the burn patient group was significantly higher than the control participants (1012.18 vs. 694.04 µg/mL, *p* < 0.005). The saliva flow rate was not measured, as the time required to provide a passive drool sample varied between participants for many different reasons, including participant age, or extent of carer support. Participants were given as much time as required to provide a minimum of 1 mL sample volume, or until they no longer wanted to provide a sample (up to ∼5 min). The increased total protein concentration in the burn samples may have resulted from a reduced cephalic response [84], increased stress from being in a hospital environment [85, 86], or dehydration [87]. It may also be increased in response to the burn injury, either as a reflection of systemic healing response, or in anticipation of delivering specific proteins to the wound site for healing (e.g., licking the wound [74]).

Several limitations of this study may have biased the data. One of the most influential limitations is the lack of a large, clinically diverse cohort of patients with burns. Due to the limited patient recruitment timeframe, there was a limited sample size and therefore statistical power. This study was not sufficiently statistically powered to detect statistically significant differences when corrections for multiple testing were applied. Due to the potential for type I and type II errors, insights from this pilot study may not be conclusive, and any trends observed should be considered with caution. This study serves as a preliminary investigation into the effect of different burn characteristics on salivary proteome to direct further research and validation in larger cohorts. Many samples initially collected were later unable to be analysed due to low sample volume. Future studies should aim to collect more than a minimum 1 mL of passive drool sample. Another drawback of this study is the lack of temporal investigation of the salivary proteome in burn patients. Proteomic evaluation of the saliva at multiple timepoints across the healing period may provide beneficial information on the healing process and may reveal biomarker candidates for prediction of healing trajectory. Additionally, protein abundance evaluated at the time of admission to hospital or shortly after burn injury, may better reflect the burn wound characteristics, as opposed to the abundance values presented here. The samples in this study were collected between 6‐ and 10‐days post‐burn because the emotional distress screening tools perform best at this time. Furthermore, a lack of collection of blood samples in this study prevents this research from being directly compared to previously published work by others which used paediatric blood samples. Having matched blood and saliva samples would enable researchers to assess whether the proteins in the saliva were indicative of local changes in the oral environment or systemic changes. Another benefit of having matched blood samples is the ability to assess the relationship between saliva and plasma abundances of specific proteins, which previous research has determined can differ [88]. Finally, the study participants were not screened for recent infections or other ailments that may have distorted their salivary protein levels, and it is possible that pro‐inflammatory processes were active in the control cohort.

Another factor which limited the identification of proteins in this study was the size of the spectral library, which contained 1310 proteins. This is smaller than other saliva protein libraries reported in the literature [89]. In this study, three different fractionation methods were used to create the spectral library, and the fractionation methods which used LDS‐PAGE electrophoresis identified less proteins than expected. Potentially, proteins were lost or not liberated effectively from the gels during the in‐gel digestion protocol, as there were large pieces of gel in each tube. Longer incubation times, increasing the tube size and increasing reagent volume may have improved this protocol. In the future, other fractionation methods such as high‐performance liquid chromatography (HPLC) [90], peptide fractionation methods [25] or combinations of several techniques may also be beneficial to reduce the complexity of samples and increase protein identification.

Future research investigating the salivary proteome in paediatric patients with burns should be performed using a more diverse patient group, include greater sample numbers, use blood to corroborate biological findings and analyse samples collected from multiple timepoints across the healing period. Additionally, validatory studies are required to investigate (1) the functional role of the proteins outlined in this work, and (2) the prognostic, diagnostic or predictive ability of these proteins with regards to burns management and treatment.

## 6 Conclusions

This pilot study was performed to investigate the salivary protein profiles of paediatric burn patients and evaluate saliva as a medium for investigating burn characteristic or clinical outcome‐related alterations in biology. Using SWATH‐MS, the relative abundance of 742 proteins was quantified in paediatric saliva. Evaluation of the differential protein abundance profiles between children with burns and healthy controls revealed that the biological response to burns could be detected using saliva and the immune/inflammatory response was elevated in children with burn injury. Furthermore, evaluation of the salivary proteome of patients with different burn characteristics revealed distinct proteins that were identified to be differentially abundant between patients with burns from different mechanisms, with different risks for developing emotional distress symptoms or that were correlated with healing time. The differentially abundant proteins identified in this study provide evidence that different burn characteristics have an impact on the response to burn injury, and that this altered response is reflected in the saliva. Overall, this study demonstrates that saliva may be a suitable replacement or supplementary medium for blood in paediatric biomarker discovery research, particularly for burns.

## Author Contributions

The manuscript was written through contributions of all authors. M.C. was involved in project and experimental design, recruitment of participants, collection of samples and clinical data, conducted experiments and performed data analysis. T.Z. assisted with conducting experiments and performing data analysis. T.P., C.P. and J.V. were involved in project and experimental design. L.C. was involved in project and experimental design, recruitment of participants and collection of samples. All authors have given approval to the final version of the manuscript.

## Conflicts of Interest

The authors declare no conflicts of interest.

## Supporting information



Supporting Information

Supporting Information

## Data Availability

The mass spectrometry proteomics data have been deposited to the PRIDE Archive (http://www.ebi.ac.uk/pride/archive/) via the PRIDE partner repository with the data set identifier PXD028078 [29, 30, 31].
