# Supplementary material for: Salivary Proteome Is Altered in Children With Small Area Thermal Burns
Source: Proteomics Clin Appl. 2025 Feb 2;19(2):e202300107. doi: 10.1002/prca.202300107 (PMC11895759; doi:10.1002/prca.202300107)
Supplement: Supplementary file 1 — Supporting Information [file PRCA-19-e202300107-s002.docx]

**Supporting Information**

**Salivary proteome is altered in children with small area thermal burns.**

**Morgan Carlton ^a, b^, Tuo Zang ^a, b^, Tony J. Parker ^a^, Chamindie Punyadeera ^c, d^, Joanne Voisey ^e^, and Leila Cuttle ^a, b,^***

**^a.^ School of Biomedical Science, Faculty of Health, Queensland University of Technology, Brisbane, Queensland, Australia**

**^b.^ Centre for Children’s Burn and Trauma Research, Centre for Children’s Health Research, Queensland University of Technology, South Brisbane, Queensland, Australia**

**^c.^ Saliva and Liquid Biopsy Translational Research Team, Centre for Biomedical Technologies, School of Biomedical Sciences, Queensland University of Technology, Kelvin Grove, Queensland, Australia**

**^d.^ The School of Environment and Science, Griffith Institute for Drug Discovery (GRIDD), and Menzies Health Institute Queensland (MIHQ), Griffith University, Queensland, Australia**

**^e.^ Centre for Genomics and Personalised Health, School of Biomedical Science, Faculty of Health, Queensland University of Technology**

*Corresponding Author

Leila Cuttle, Queensland University of Technology (QUT), Faculty of Health, School of Biomedical Science, Center for Children’s Health Research (CCHR), 62 Graham Street, South Brisbane, Queensland, Australia 4101, Email: Leila.cuttle@qut.edu.au, Ph: +61730697208

**Table of Contents**

[**Supplementary Figure 1: Pipeline for creating Paediatric Saliva protein Spectral Library.** 3](#_Toc80712445)

[**Supplementary Figure 2: Three measures were used to assess the most appropriate normalisation method for the Proteomic data, including (A) Intragroup Pooled coefficient of variation, (B) Intragroup Pooled Median Absolute Deviation and (C) Intragroup Pooled Estimation of Variance.** 4](#_Toc80712446)

[**Supplementary Table 1: Twenty-nine proteins were found to have significantly different abundance between children with burns and healthy controls.** 5](#_Toc80712447)

[**Supplementary Figure 3: Eight, Eleven and four proteins were found to be robustly differentially abundant between (a) patients and controls, (B) scald and contact burns, and (C) patients with high and low risk for Emotional Distress, respectively.** 6](#_Toc80712448)

[**Supplementary Table 2: Twenty-nine proteins were found to have significantly different abundance between children with burns and healthy controls.** 7](#_Toc80712449)

[**Supplementary Table 3: Twenty-nine proteins were found to have significantly different abundance between children with burns and healthy controls.** 8](#_Toc80712450)

# **Supplementary Figure 1: Pipeline for creating Paediatric Saliva protein Spectral Library.**


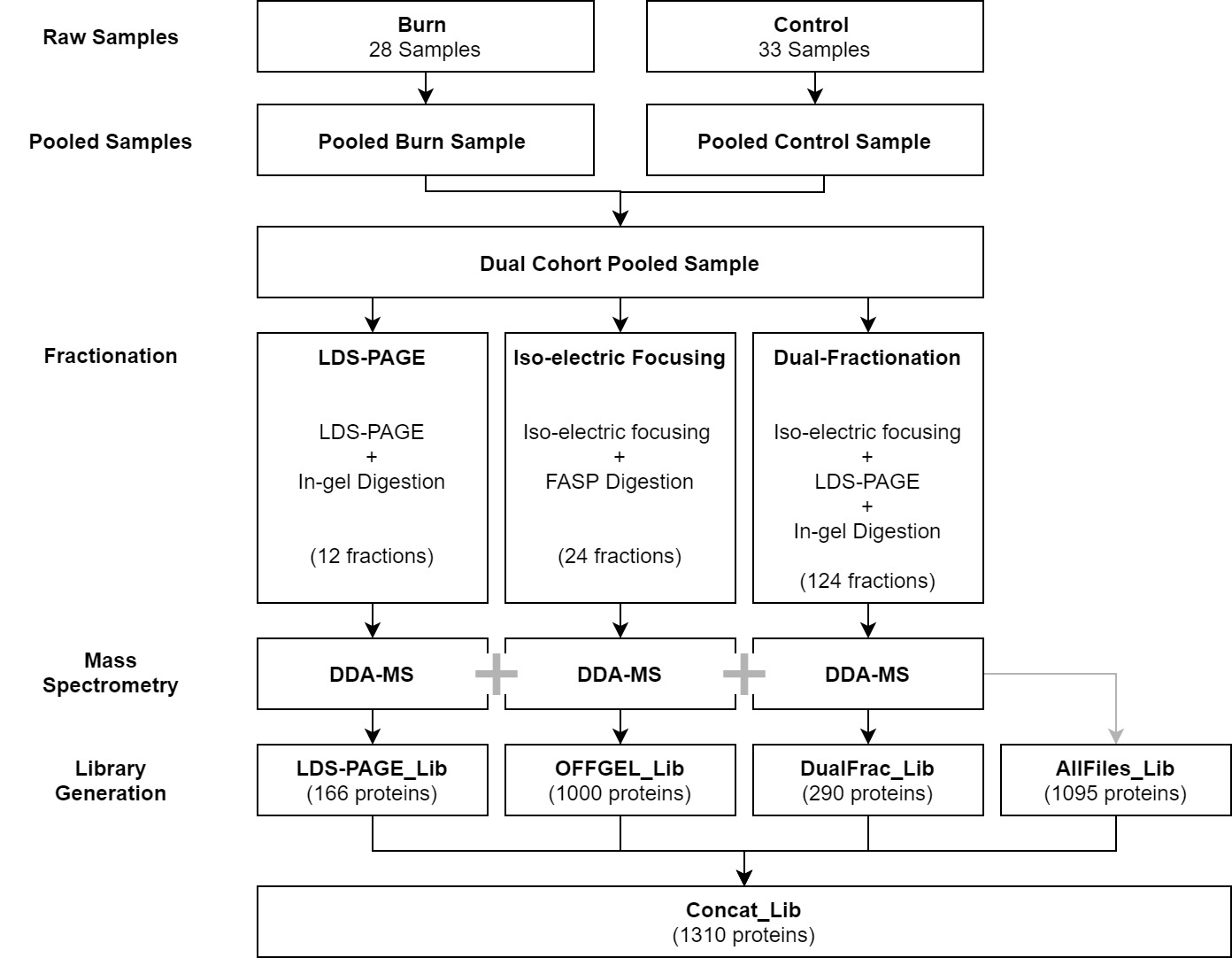


**Global Pooled Sample**


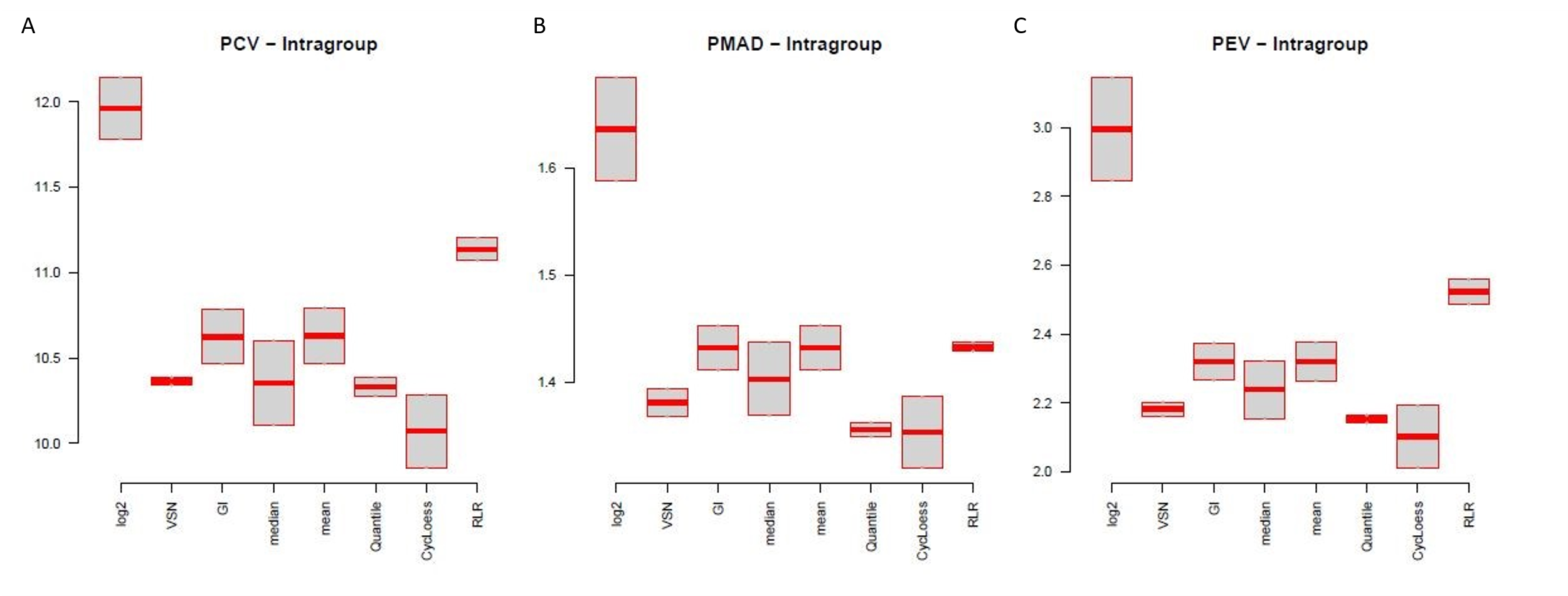


**Supplementary Figure 2: Three measures were used to assess the most appropriate normalisation method for the Proteomic data, including (A) Intragroup Pooled coefficient of variation, (B) Intragroup Pooled Median Absolute Deviation and (C) Intragroup Pooled Estimation of Variance.** Output exported from NormalyzerDE.

# **Supplementary Table 1: Twenty-nine proteins were found to have significantly different abundance between children with burns and healthy controls.**

| **Protein ID** | **Protein Name** | **Accession #** | **Mean (SD) abundance ^a^** | | **p value ^b^** |
| --- | --- | --- | --- | --- | --- |
|  |  |  | **Control** | **Burn** |  |
| ARGI1 | Arginase-1 | P05089 | 14.49 (0.54) | 15.08 (0.72) | 0.001 |
| MOES | Moesin | P26038 | 15.81 (0.91) | 15.01 (0.77) | 0.002 |
| KV229 | Immunoglobulin kappa variable 2-29 | A2NJV5 | 13.50 (1.30) | 14.69 (1.23) | 0.003 |
| DESP | Desmoplakin | P15924 | 16.34 (1.40) | 17.64 (1.70) | 0.003 |
| RBM12 | RNA-binding protein 12 | Q9NTZ6 | 11.77 (1.89) | 10.40 (1.09) | 0.005 |
| NGAL | Neutrophil gelatinase-associated lipocalin | P80188 | 19.54 (0.91) | 18.60 (1.18) | 0.006 |
| T132A | Transmembrane protein 132A | Q24JP5 | 7.83 (3.64) | 9.94 (1.61) | 0.008 |
| KAP0 | cAMP-dependent protein kinase type I-alpha regulatory subunit | P10644 | 10.65 (1.07) | 9.77 (0.93) | 0.008 |
| LAMP2 | Lysosome-associated membrane glycoprotein 2 | P13473 | 14.30 (1.05) | 13.51 (0.91) | 0.009 |
| K2C3 | Keratin, type II cytoskeletal 3 | P12035 | 13.86 (0.67) | 14.59 (1.39) | 0.011 |
| PTN6 | Tyrosine-protein phosphatase non-receptor type 6 | P29350 | 10.66 (1.58) | 9.56 (0.96) | 0.013 |
| PKP1 | Plakophilin-1 | Q13835 | 11.48 (1.20) | 10.79 (1.09) | 0.014 |
| RPE | Ribulose-phosphate 3-epimerase | Q96AT9 | 13.40 (1.31) | 12.78 (1.04) | 0.014 |
| PA2GA | Phospholipase A2, membrane associated | P14555 | 9.99 (1.18) | 11.23 (1.98) | 0.016 |
| KRA61 | Keratin-associated protein 6-1 | Q3LI64 | 10.09 (1.82) | 11.57 (1.98) | 0.017 |
| GSHR | Glutathione reductase, mitochondrial | P00390 | 12.92 (1.18) | 12.19 (0.96) | 0.017 |
| LV319 | Immunoglobulin lambda variable 3-19 | P01714 | 13.93 (1.20) | 13.30 (0.86) | 0.021 |
| SPB4 | Serpin B4 | P48594 | 12.26 (0.86) | 12.77 (0.69) | 0.022 |
| K2C1B | Keratin, type II cytoskeletal 1b | Q7Z794 | 12.19 (1.64) | 12.93 (2.12) | 0.028 |
| B2MG | Beta-2-microglobulin | P61769 | 18.29 (1.34) | 19.36 (1.47) | 0.029 |
| TSN6 | Tetraspanin-6 | O43657 | 11.88 (1.23) | 12.43 (1.42) | 0.029 |
| SMR3B | Submaxillary gland androgen-regulated protein 3B | P02814 | 15.70 (2.21) | 18.06 (3.05) | 0.034 |
| DSG3 | Desmoglein-3 | P32926 | 15.37 (0.78) | 16.01 (0.88) | 0.036 |
| TBA1C | Tubulin alpha-1C chain | Q9BQE3 | 12.60 (1.40) | 11.93 (1.22) | 0.038 |
| CATA | Catalase | P04040 | 17.58 (1.24) | 16.65 (1.11) | 0.039 |
| K22O | Keratin, type II cytoskeletal 2 oral | Q01546 | 15.48 (1.31) | 16.71 (1.85) | 0.04 |
| S10AB | Protein S100-A11 | P31949 | 14.82 (1.43) | 14.38 (0.96) | 0.044 |
| TKT | Transketolase | P29401 | 18.63 (0.70) | 18.14 (0.57) | 0.048 |
| KLK10 | Kallikrein-10 | O43240 | 15.73 (1.08) | 15.07 (0.97) | 0.049 |
| ^a.^ Calculated from CycLoess normalised data. ^b.^ Significance determined using Multivariate General Linear Model. | | | | | |

**
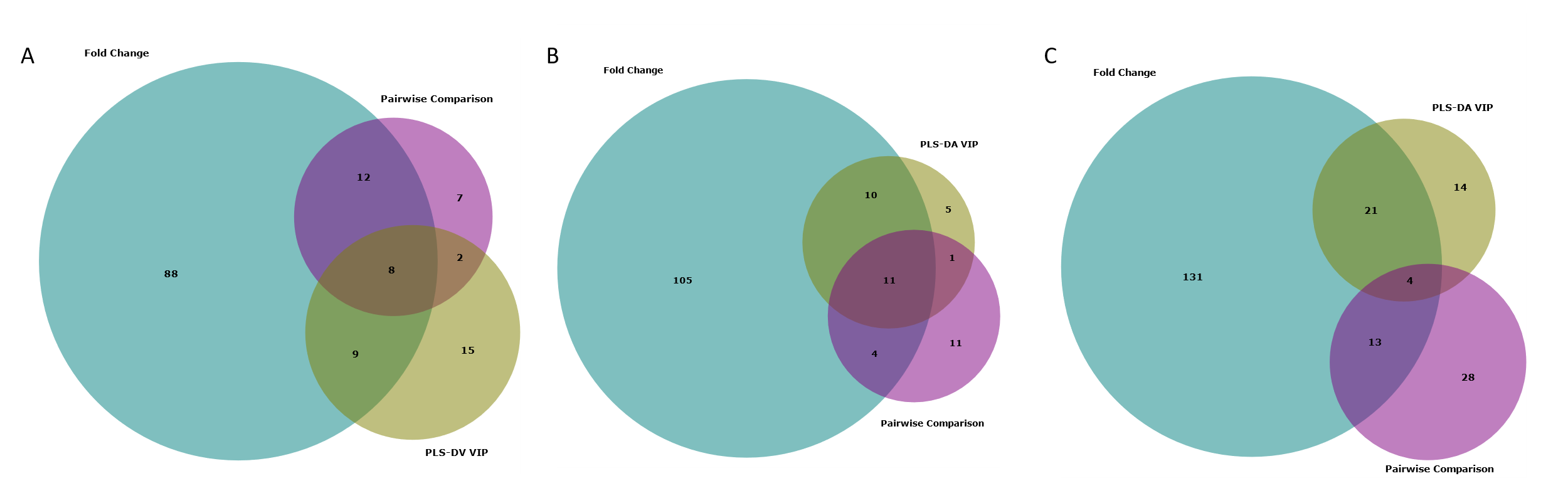
**

# **Supplementary Figure 3: Eight, Eleven and four proteins were found to be robustly differentially abundant between (a) patients and controls, (B) scald and contact burns, and (C) patients with high and low risk for Emotional Distress, respectively.**

| **Protein ID** | **Protein Name** | **Accession #** | **Mean Normalised Abundance ^a^** | | **p value ^b^** |  |
| --- | --- | --- | --- | --- | --- | --- |
|  |  |  | **Scald** | **Contact** |  |  |
| SPIT1 | Kunitz-type protease inhibitor 1 | O43278 | 14.78 (0.39) | 15.62 (0.46) | 0.000 |  |
| HDHD2 | Haloacid dehalogenase-like hydrolase domain-containing protein 2 | Q9H0R4 | 12.63 (1.39) | 10.63 (1.22) | 0.003 |  |
| CALL3 | Calmodulin-like protein 3 | P27482 | 16.00 (0.71) | 17.28 (1.01) | 0.004 |  |
| C1RL | Complement C1r subcomponent-like protein | Q9NZP8 | 12.35 (1.33) | 10.58 (1.25) | 0.006 |  |
| SH3L1 | SH3 domain-binding glutamic acid-rich-like protein | O75368 | 13.71 (1.03) | 15.05 (1.04) | 0.009 |  |
| HV313 | Immunoglobulin heavy variable 3-13 | P01766 | 14.50 (1.33) | 13.14 (0.71) | 0.010 |  |
| CHIT1 | Chitotriosidase-1 | Q13231 | 14.37 (0.88) | 15.39 (0.75) | 0.012 |  |
| SBP1 | Methanethiol oxidase | Q13228 | 12.05 (0.76) | 11.14 (0.78) | 0.016 |  |
| LV218 | Immunoglobulin lambda variable 2-18 | A0A075B6J9 | 13.80 (1.23) | 11.74 (2.20) | 0.019 |  |
| K1C24 | Keratin, type I cytoskeletal 24 | Q2M2I5 | 12.07 (1.58) | 13.55 (0.94) | 0.020 |  |
| CTL4 | Choline transporter-like protein 4 | Q53GD3 | 12.68 (1.65) | 10.76 (1.71) | 0.020 |  |
| CO4B | Complement C4-B | P0C0L5 | 17.78 (0.54) | 16.78 (1.15) | 0.022 |  |
| NICA | Nicastrin | Q92542 | 12.50 (1.14) | 14.07 (1.60) | 0.022 |  |
| PKP1 | Plakophilin-1 | Q13835 | 10.81 (0.81) | 11.88 (1.10) | 0.023 |  |
| HV373 | Immunoglobulin heavy variable 3-73 | A0A0B4J1V6 | 12.31 (1.77) | 10.44 (1.66) | 0.025 |  |
| KVD12 | Immunoglobulin kappa variable 1D-12 | P01611 | 11.95 (1.94) | 10.21 (1.20) | 0.026 |  |
| GRAN | Grancalcin | P28676 | 12.63 (0.74) | 11.74 (0.89) | 0.026 |  |
| K1C13 | Keratin, type I cytoskeletal 13 | P13646 | 15.83 (0.91) | 17.61 (2.15) | 0.027 |  |
| SPR1A | Cornifin-A | P35321 | 14.45 (1.22) | 16.79 (2.90) | 0.03 |  |
| K2C6A | Keratin, type II cytoskeletal 6A | P02538 | 10.62 (1.56) | 12.99 (2.80) | 0.031 |  |
| PROS | Vitamin K-dependent protein S | P07225 | 13.37 (1.19) | 11.89 (1.64) | 0.032 |  |
| HV372 | Immunoglobulin heavy variable 3-72 | A0A0B4J1Y9 | 15.15 (1.11) | 14.01 (1.11) | 0.033 |  |
| LV469 | Immunoglobulin lambda variable 4-69 | A0A075B6H9 | 6.38 (5.24) | 10.62 (2.82) | 0.037 |  |
| LBP | Lipopolysaccharide-binding protein | P18428 | 12.22 (1.57) | 10.48 (1.89) | 0.039 |  |
| PTPRF | Receptor-type tyrosine-protein phosphatase F | P10586 | 11.48 (1.14) | 10.36 (1.16) | 0.043 |  |
| ^a.^ Calculated from CycLoess normalised data. ^b.^ Significance determined using Multivariate General Linear Model. | | | | | | |

# **Supplementary Table 2: Twenty-seven proteins were determined to be significantly different in abundance between patients with scald burns and patients with contact burns.**

# **Supplementary Table 3: Forty-five proteins were determined to be significantly different in abundance between patients categorised as low risk for the development of emotional distress symptoms and patients categorised as high risk.**

| **Protein ID** | **Protein Name** | **Accession #** | **Mean Normalised Abundance ^a^** | | ***p* value ^b^** |
| --- | --- | --- | --- | --- | --- |
|  |  |  | **Low Risk** | **High Risk** |  |
| PP1A | Serine/threonine-protein phosphatase PP1-alpha catalytic subunit | P62136 | 13.64 (0.78) | 12.11 (0.50) | 0.000 |
| APOB | Apolipoprotein B-100 | P04114 | 16.32 (1.01) | 14.64 (0.65) | 0.001 |
| TACD2 | Tumor-associated calcium signal transducer 2 | P09758 | 12.43 (1.21) | 10.70 (0.79) | 0.003 |
| PRDX1 | Peroxiredoxin-1 | Q06830 | 15.65 (1.27) | 17.39 (0.82) | 0.004 |
| FABP5 | Fatty acid-binding protein 5 | Q01469 | 16.96 (0.86) | 18.10 (0.60) | 0.005 |
| CAH6 | Carbonic anhydrase 6 | P23280 | 20.93 (0.94) | 19.77 (0.85) | 0.012 |
| PITH1 | PITH domain-containing protein 1 | Q9GZP4 | 13.71 (0.96) | 12.61 (0.60) | 0.012 |
| HDHD2 | Haloacid dehalogenase-like hydrolase domain-containing protein 2 | Q9H0R4 | 12.17 (1.49) | 10.33 (1.36) | 0.012 |
| LYPD3 | Ly6/PLAUR domain-containing protein 3 | O95274 | 16.68 (0.79) | 17.85 (1.24) | 0.014 |
| PROM1 | Prominin-1 | O43490 | 12.58 (1.31) | 14.12 (1.17) | 0.015 |
| PEBP1 | Phosphatidylethanolamine-binding protein 1 | P30086 | 16.62 (1.13) | 18.00 (1.15) | 0.015 |
| FIBB | Fibrinogen beta chain | P02675 | 18.69 (1.46) | 17.22 (0.33) | 0.017 |
| MUC5B | Mucin-5B | Q9HC84 | 19.74 (2.06) | 21.90 (1.00) | 0.017 |
| LACRT | Extracellular glycoprotein lacritin | Q9GZZ8 | 12.74 (1.35) | 15.50 (3.76) | 0.018 |
| CATS | Cathepsin S | P25774 | 13.46 (0.76) | 14.37 (0.80) | 0.019 |
| KRA32 | Keratin-associated protein 3-2 | Q9BYR7 | 11.24 (1.45) | 9.73 (0.81) | 0.019 |
| KRA24 | Keratin-associated protein 2-4 | Q9BYR9 | 14.07 (1.26) | 12.76 (0.73) | 0.019 |
| AMY1A | Alpha-amylase 1A | P0DUB6 | 24.25 (1.12) | 22.72 (1.72) | 0.020 |
| HTRA1 | Serine protease HTRA1 | Q92743 | 11.80 (1.51) | 13.54 (1.51) | 0.021 |
| PPIB | Peptidyl-prolyl cis-trans isomerase B | P23284 | 18.65 (1.29) | 17.31 (0.94) | 0.024 |
| NGAL | Neutrophil gelatinase-associated lipocalin | P80188 | 19.83 (0.72) | 18.92 (1.01) | 0.024 |
| A1BG | Alpha-1B-glycoprotein | P04217 | 19.24 (1.39) | 17.88 (0.78) | 0.026 |
| K2C3 | Keratin, type II cytoskeletal 3 | P12035 | 14.07 (0.56) | 13.42 (0.69) | 0.028 |
| UB2V1 | Ubiquitin-conjugating enzyme E2 variant 1 | Q13404 | 12.02 (1.64) | 13.57 (0.94) | 0.032 |
| QORX | Quinone oxidoreductase PIG3 | Q53FA7 | 12.65 (0.98) | 11.66 (0.83) | 0.032 |
| GOLM1 | Golgi membrane protein 1 | Q8NBJ4 | 13.94 (1.31) | 15.29 (1.24) | 0.033 |
|  | | | | | |

| **Supplementary Table 3** Continued. | | | | | |
| --- | --- | --- | --- | --- | --- |
| **Protein ID** | **Protein Name** | **Accession #** | **Mean Normalised Abundance ^a^** | | ***p* value ^b^** |
|  |  |  | **Low Risk** | **High Risk** |  |
| ASC | Apoptosis-associated speck-like protein containing a CARD | Q9ULZ3 | 12.39 (0.96) | 13.40 (0.98) | 0.034 |
| 1433B | 14-3-3 protein beta/alpha | P31946 | 13.85 (0.96) | 14.90 (1.16) | 0.036 |
| PRR27 | Proline-rich protein 27 | Q6MZM9 | 18.69 (1.86) | 16.93 (1.30) | 0.036 |
| LMNA | Prelamin-A/C | P02545 | 13.44 (1.33) | 12.05 (1.44) | 0.037 |
| LCN1 | Lipocalin-1 | P31025 | 18.99 (2.76) | 22.13 (3.69) | 0.037 |
| H14 | Histone H1.4 | P10412 | 13.34 (0.74) | 12.61 (0.66) | 0.038 |
| SAP3 | Ganglioside GM2 activator | P17900 | 13.95 (0.83) | 14.77 (0.77) | 0.039 |
| MTMR2 | Myotubularin-related protein 2 | Q13614 | 13.07 (1.49) | 11.48 (1.76) | 0.039 |
| FIBG | Fibrinogen gamma chain | P02679 | 18.33 (1.51) | 17.04 (0.45) | 0.040 |
| DSG3 | Desmoglein-3 | P32926 | 15.15 (0.77) | 15.86 (0.55) | 0.041 |
| CFAB | Complement factor B | P00751 | 17.46 (1.09) | 16.44 (0.87) | 0.042 |
| MSLN | Mesothelin | Q13421 | 13.20 (1.29) | 14.51 (1.42) | 0.043 |
| CO3 | Complement C3 | P01024 | 19.56 (1.32) | 18.35 (0.98) | 0.044 |
| IGLC3 | Immunoglobulin lambda constant 3 | P0DOY3 | 14.49 (1.79) | 16.37 (2.16) | 0.044 |
| CLUS | Clusterin | P10909 | 17.34 (1.32) | 16.18 (0.75) | 0.044 |
| CALL3 | Calmodulin-like protein 3 | P27482 | 16.24 (0.96) | 17.22 (1.11) | 0.045 |
| TGM3 | Protein-glutamine gamma-glutamyltransferase E | Q08188 | 20.10 (1.15) | 18.97 (1.17) | 0.045 |
| BT1A1 | Butyrophilin subfamily 1 member A1 | Q13410 | 10.93 (1.44) | 12.77 (2.71) | 0.048 |
| HPRT | Hypoxanthine-guanine phosphoribosyltransferase | P00492 | 13.84 (1.44) | 12.39 (1.24) | 0.034 |

| ^a.^ Calculated from CycLoess normalised data. ^b.^ Significance determined using Multivariate General Linear Model. |
| --- |
